# Supplementary material for: Epigallocatechin-3-gallate modulates Tau Post-translational modifications and cytoskeletal network
Source: Oncotarget. 2021 May 25;12(11):1083–99. doi: 10.18632/oncotarget.27963 (PMC8169072; doi:10.18632/oncotarget.27963)
Supplement: Supplementary file 1 [file oncotarget-12-1083-s001.pdf]

Epigallocatechin-3-gallate modulates Tau Post-translational modifications and cytoskeletal network

SUPPLEMENTARY MATERIALS

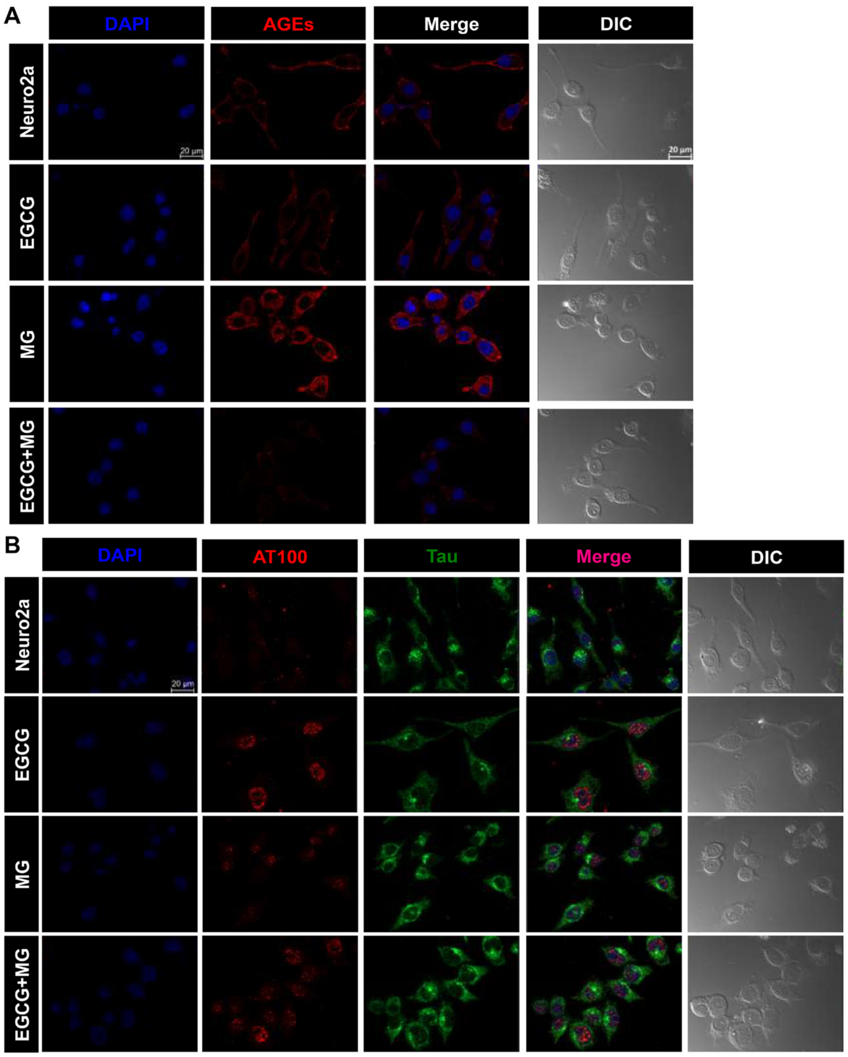

Supplementary Figure 1: MG-induced glycation and Tau phosphorylation. (**A**) Single channel images showing increase in AGEs on MG treatment in neuro2a cells and their inhibition by EGCG. Neuro2a cells untreated and EGCG treated show neuritic extensions and intact cell shape for both modifications. MG treatment alters the cell morphology resulting in loss neuritic extensions and rounding off of cells. Supplementation of EGCG with MG shows normal cell morphology with maintained cell shape and neuritic extensions. (**B**) AT 100 localization on EGCG treatment surrounding nuclear periphery which is disrupted by MG treatment.

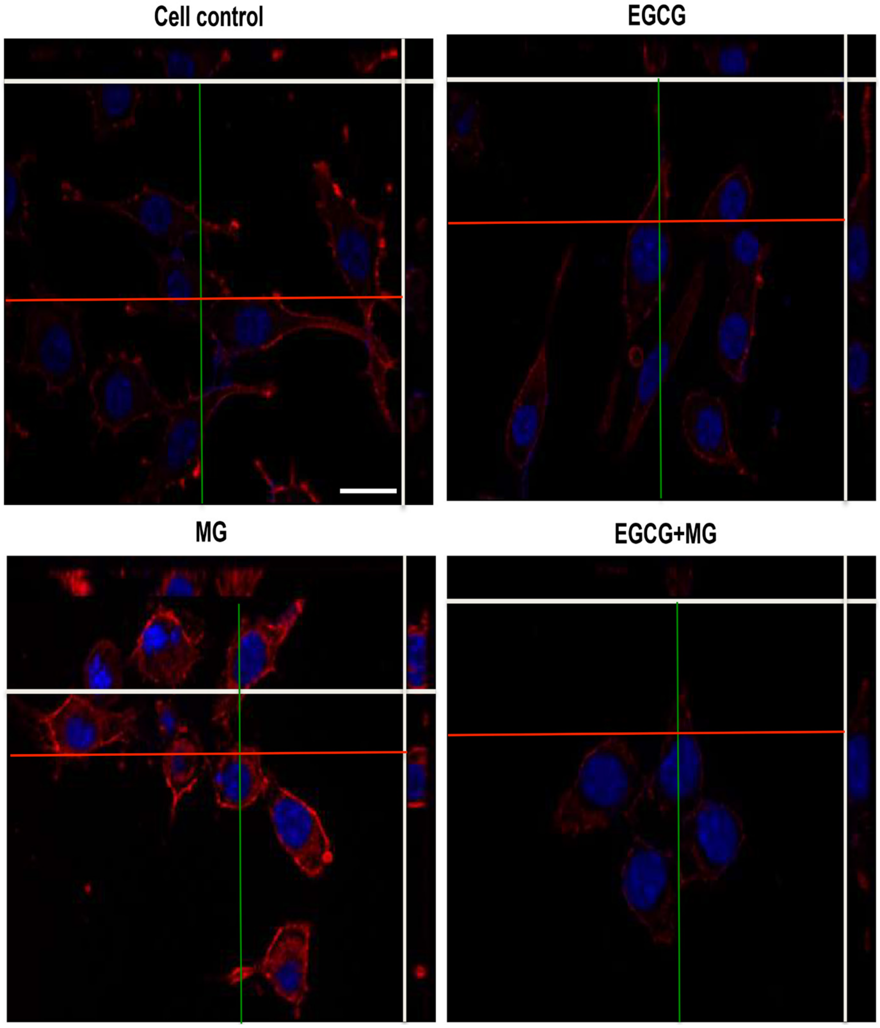

Supplementary Figure 2: Orthogonal projection analysis of MG-induced AGEs positive neuro2a cells. Orthogonal projectional analysis shows basal level of AGEs in untreated and EGCG treated cells. MG treatment induces global glycation in the cells resulting in enhanced AGEs formation in the cells. EGCG is found to inhibit this effect of MG and reduce the global AGEs formation in the cells. Scale bar: 20 μm.

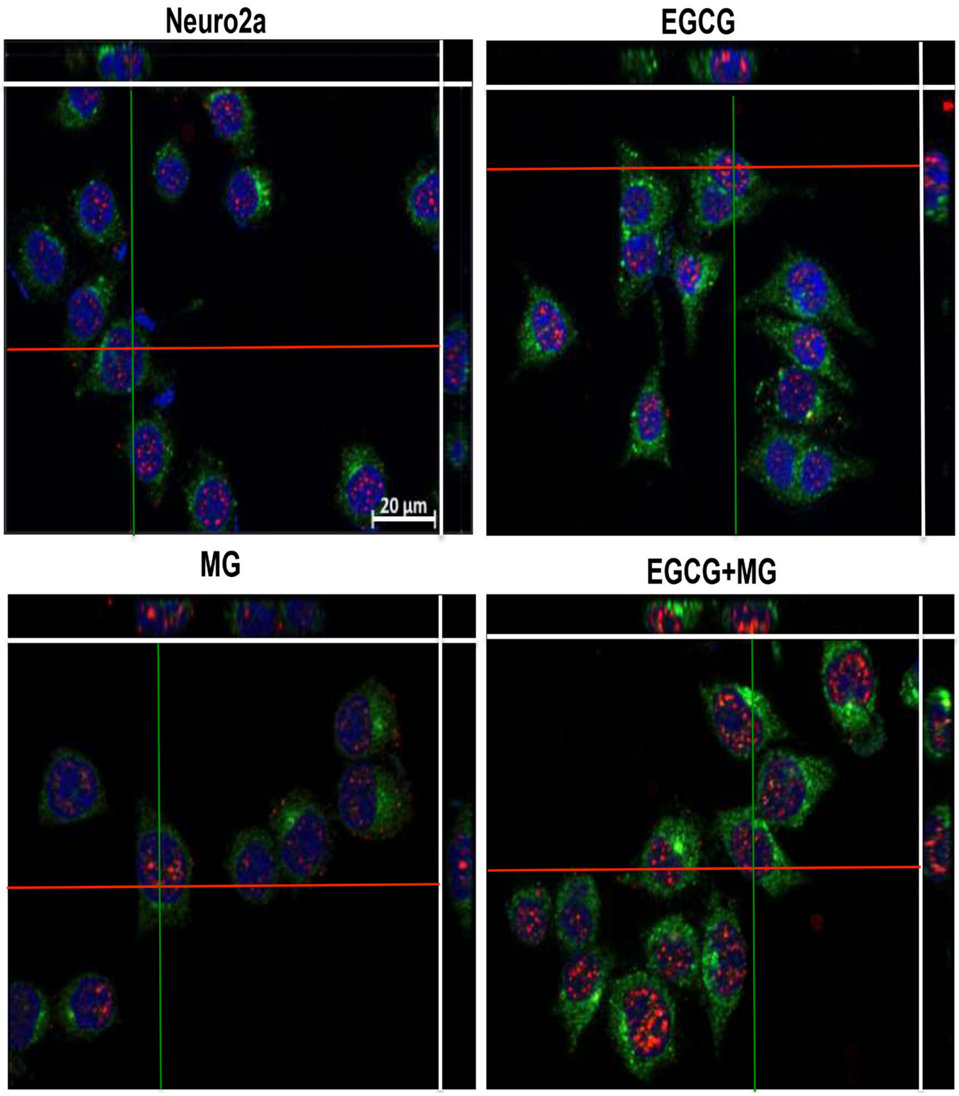

Supplementary Figure 3: Orthogonal projection analysis of MG-induced Tau phosphorylation in neuro2a cells. AT100 phospho-Tau is present at basal levels in control cells and distributed throughout the cytoplasm and nucleus. EGCG treatment is found to change the localization of phospho-Tau in the nucleus at the periphery in a ring like manner. MG treatment disrupts this arrangement in as seen in the orthogonal projections whereas MG and EGCG together maintain the AT100 phospho-Tau in the nucleus.

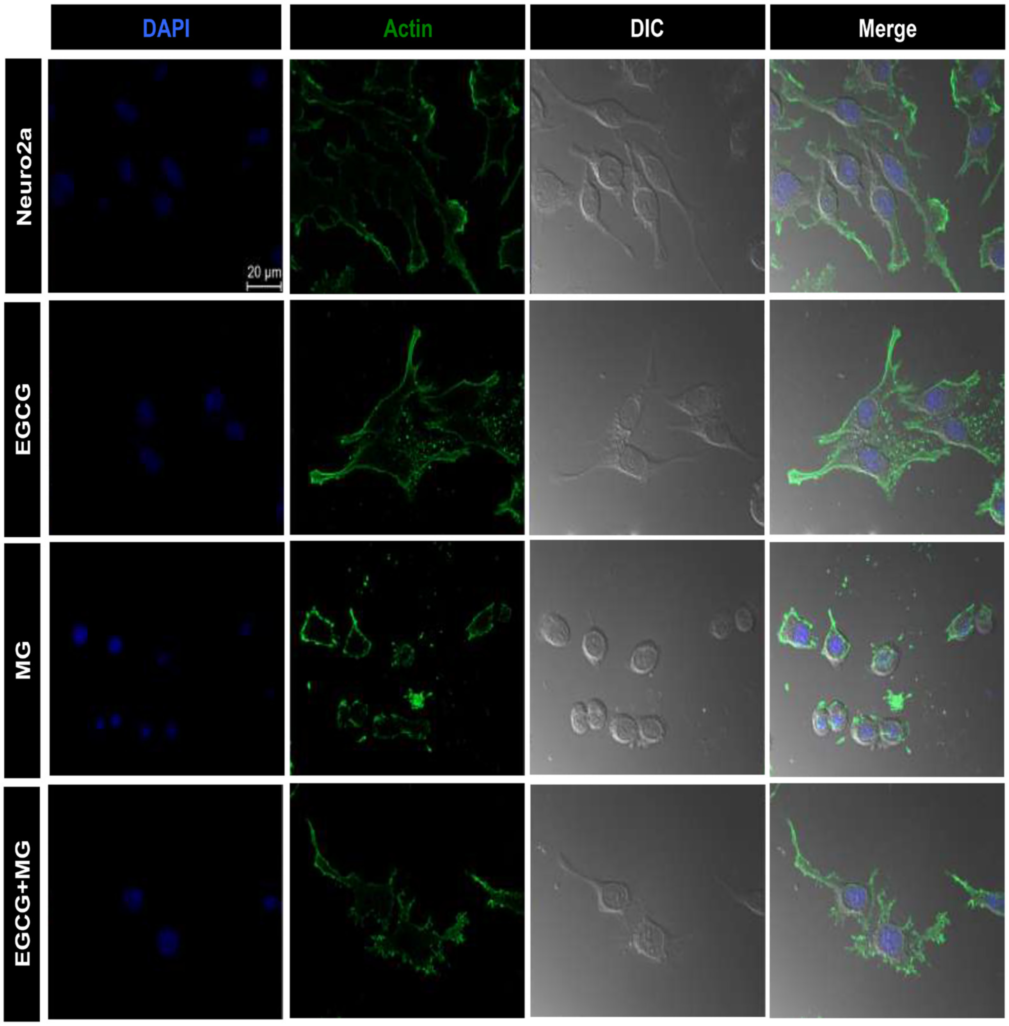

Supplementary Figure 4: MG-induced disruption of actin cytoskeleton. Untreated neuro2a cells show visible neuritic extensions rich in actin also evidenced by merge of DIC with actin. EGCG treated cells more of minute neuritic extensions along with the long extensions. MG treatment severely disrupts actin cytoskeleton and neuritic extensions (merge). EGCG treatment with MG leads to formation of both minute and long actin rich neuritic extensions.
